# Resveratrol improves human umbilical cord-derived mesenchymal stem cells repair for cisplatin-induced acute kidney injury

**DOI:** 10.1038/s41419-018-0959-1

**Published:** 2018-09-20

**Authors:** Rongxue Zhang, Lei Yin, Bin Zhang, Hui Shi, Yaoxiang Sun, Cheng Ji, Jingyan Chen, Peipei Wu, Leilei Zhang, Wenrong Xu, Hui Qian

**Affiliations:** 1 Zhenjiang Key Laboratory of High Technology Research on Exosomes Foundation and Transformation Application, Jiangsu Key Laboratory of Medical Science and Laboratory Medicine, School of Medicine, Jiangsu University, Zhenjiang, Jiangsu People’s Republic of China; 2 Huai’an maternity and child health care hospital, Huai’an, Jiangsu People’s Republic of China; 3 Key Laboratory of Embryo Molecular Biology, Ministry of Health and Shanghai Key Laboratory of Embryo and Reproduction Engineering, Shanghai, People’s Republic of China

## Abstract

Human umbilical cord-derived mesenchymal stem cells (hucMSCs) are a promising tool for damaged tissues repair, especially for the kidney. However, their efficacy requires improvement. In order to optimize the clinical utility of hucMSCs, we adopted a strategy of treating hucMSCs with 20 μmol/L of resveratrol (Res-hucMSCs), applying it in a cisplatin-induced acute kidney injury model. Interestingly, we found that Res-hucMSCs exhibited a more efficient repairing effect than did hucMSCs. Resveratrol-promoted hucMSCs secreted platelet-derived growth factor-DD (PDGF-DD) into renal tubular cells resulting in downstream phosphorylation of extracellular signal-regulated kinase (ERK), which inhibited renal tubular cells apoptosis. In contrast, PDGF-DD knockdown impaired the renal protection of Res-hucMSCs. In addition, angiogenesis induced by PDGF-DD in endothelial cells was also involved in the renal protection of Res-hucMSCs. The conditioned medium of Res-hucMSCs accelerated proliferation and migration of vascular endothelial cells in vitro and CD31 was in a high-level expression in Res-hucMSCs group in vivo. Nevertheless, the angiogenesis was abrogated when Res-hucMSCs were treated with PDGF-DD siRNA. In conclusion, our findings showed that resveratrol-modified hucMSCs activated ERK pathway in renal tubular cells and promoted angiogenesis in endothelial cells via paracrine PDGF-DD, which could be a novel strategy for enhancing the therapy efficacy of hucMSCs in cisplatin-induced kidney injury.

## Introduction

Acute kidney injury (AKI) is a frequent clinical syndrome, which is characterized by a sudden loss of the kidney function^1^. AKI is caused by a variety of factors, including surgery, hypoxia, drugs, mechanical trauma, inflammation, cardiopulmonary bypass, and hemodynamic instability^2^. At present, although remarkable progress has been made in dialysis and renal replacement therapy, the morbidity and mortality of patients with AKI remain high^3,4^. Therefore, patients with AKI urgently need a new therapy strategy.

Mesenchymal stem cells (MSCs) are a promising tool for the treatment of kidney injury^5,6^. MSCs can be isolated from the bone marrow, umbilical cord, adipose tissues, and other adult tissues. Lower immunogenicity and easier availability turn hucMSCs into a favorable candidate for injured tissue repair^7^. Although previous studies showed that hucMSCs can alleviate AKI or chronic kidney injury^8,9^, the efficacy of stem cell-based therapy can be further improved. Small-molecule drugs have an important role in regulating stem cell fate and function, and facilitate the development of cell-based therapies^10^. For example, resveratrol (Res, 3,5,4’-trihydroxy-trans-stilbene)-modified cardiac stem cells exerted an improved impairing effect on infarcted myocardium by increasing the survival and engraftment of implanted cardiac stem cells^11^.

Res, a natural polyphenolic compound, is derived from several plants such as grapes, peanuts, and mulberries. Res is reported to have various biologic functions including anti-inflammatory, antioxidant, anti-aging, and so on^12^. Based on these biologic functions, Res has been widely investigated in regenerative medicine. It was reported that Res alleviated multiple organs damage, particularly in the kidney^13,14^. In addition, Res could protect MSCs against inflammation and oxidative injury^15,16^. However, the effect of Res on MSCs-based therapy has not been investigated. It remains unknown whether Res-modified hucMSCs can show a more efficient repairing ability than did hucMSCs in tissue injury.

Here we investigated the effect of Res-hucMSCs on cisplatin-induced AKI. Our findings demonstrated that hucMSCs primed with Res activated ERK signal pathway in renal tubular cells and promoted angiogenesis in endothelial cells via paracrine platelet-derived growth factor-DD (PDGF-DD), which preferably inhibited renal tubular cell apoptosis. Res-hucMSCs have a higher efficiency than did hucMSCs in the repair of cisplatin-induced AKI.

## Materials and methods

### Cell culture

All experiment protocols were approved by the medical ethics committee of Jiangsu University (2012258). Fresh human umbilical cords were obtained from consenting mothers in the affiliated hospital of Jiangsu University. HucMSCs were isolated as described previously^17^ and cultured in MEM Alpha basic (α-MEM, Gibco) with 10% fetal bovine serum (FBS, Excell), penicillin and streptomycin (Gibco). The cells in passages 3–6 were used for additional experiments. Rat renal tubular epithelial cell lines (NRK-52E) and human umbilical vein endothelial cell (HUVEC) were purchased from Cell Bank (Chinese Academy of Sciences, Shanghai, China) and maintained in high-glucose Dulbecco’s modified Eagle’s medium (DMEM, Gibco) containing 10% FBS.

### Preparation of Res-hucMSCs

Res (Sigma) was dissolved in dimethyl sulfoxide (DMSO) to prepare a 20 mmol/L stock solution. In the following experiments, the concentration and time of Res treating hucMSCs were 20 μmol/L and 12 h. HucMSCs treated with 0.1% DMSO (DMSO-hucMSCs) acted as the control. The conditioned medium (CdM) referred to the cell supernatant of culturing hucMSCs for 24 h after with or without Res treatment.

### Animal model of AKI

Adult female Sprague–Dawley rats weighing 180–220 g were purchased from the Laboratory Animal Center of Jiangsu University and randomly divided into several groups (*n* = 6/group). The AKI rat model was established as described previously^18^. After 6 mg/kg cisplatin intraperitoneal injection for 24 h, 1 × 10^6^ hucMSCs (with or without Res treatment) were transplanted via tail vein. Phosphate-buffered saline (PBS)-injected rats served as a control. All animals were killed at Day 5 after cisplatin injection. Renal function, histological changes, and tubular apoptosis were evaluated.

### Cell labeling and tracing

HucMSCs (1 × 10^6^/mL) with or without Res treatment were incubated with 5 μL of the membrane dye DiR (Thermo Fisher Scientific) at 37 °C for 1 h. The unbound DiR was removed by washing with PBS and the labeled cells were resuspended in PBS and transplanted into AKI rats via tail vein. In-Vivo Imaging System (IVIS Spectrum, PE) was used to observe the engraftment of infused hucMSCs (with and without Res treatment) in renal tissues at 24 h or 96 h after hucMSCs injection.

### In vitro experiments

Rat renal tubular epithelial cell line NRK-52E was used to do experiments in vitro. Experiments were divided in four groups: control (without cisplatin treatment), cisplatin (7.5 μmol/L cisplatin treatment for 12 h), DMSO-hucMSCs (7.5 μmol/L cisplatin treatment for 12 h and DMSO-hucMSCs co-culturing for 36 h), and Res-hucMSCs (7.5 μmol/L cisplatin treatment for 12 h and Res-hucMSCs co-culturing for 36 h).

### Western blotting

Kidney tissues or cells were lysed in a radioimmunoprecipitation assay buffer containing proteinase inhibitors (phenylmethylsulfonyl fluoride, Pierce). Protein samples were separated by SDS-polyacrylamide gel electrophoresis, transferred to the polyvinylidene difluoride membrane (Millipore), blocked in 5% skim milk, and incubated with primary antibodies and horseradish peroxidase-conjugated secondary antibodies (Invitrogen). Primary antibodies used in this study were as following: c-IAP1 (CST), Bcl-xl (SAB), Bax (Bioworld), Actived-caspase3 (Bioworld), p-ERK (CST), ERK (CST), PDGF-DD (Santa Cruz), PDGFR-β (Bioworld), and β-actin (Bioworld).

### Quantitative reverse-transcriptase PCR

Total RNA of kidney tissues and cells was extracted by using the Trizol reagent (Invitrogen). One microgram of RNA was reverse transcribed to synthesized cDNA according to the manufacturer’s instructions (Vazyme). PCR was performed using QuantiTect SYBR Green PCR kit (CWBIO). The primer sequences of genes are listed in Table 1.

**Table 1:** Sequences of qRT-PCR primersPer style, gene names should be italics. Hence, please mark for italicization instances of gene names.Thank you for your advice. We have modified gene names to Italic in proof.

Gene (human)	Primer	Sequences (5′–3′)	Annealing temperature	Fragment size (bp)
*PDGF-DD*	Forward primer	GAACAGCTACCCCAGGAACC	60 °C	193
	Reverse primer	CTTGTGTCCACACCATCGTC		
*PDGF-BB*	Forward primer	CCATTCCCGAGGAGCTTTATG	60 °C	125
	Reverse primer	GGTCATGTTCAGGTCCAACTC		
*HGF*	Forward primer	ATGCATGACCTGCAATGGG	60 °C	191
	Reverse primer	GAGTATAGCACCATGGCCTCG		
*bFGF*	Forward primer	AGAAGAGCGACCCTCACATCA	60 °C	82
	Reverse primer	CGGTTAGCACACACTCCTTTG		
*VEGF*	Forward primer	CCTTGCTGCTCTACCTCCAC	60 °C	280
	Reverse primer	ATCTGCATGGTGATGTTGGA		
*Nanog*	Forward primer	CCTGATTCTTCCACCAGTCC	60 °C	292
	Reverse primer	TGCTATTCTTCGGCCAGTTG		
*Sox2*	Forward primer	ACACCAATCCCATCCACACT	60 °C	224
	Reverse primer	GCAAACTTCCTGCAAAGCTC		
*Sall4*	Forward primer	TCGATGGCCAACTTCCTTC	60 °C	142
	Reverse primer	GAGCGGACTCACACTGGAGA		
*β-Actin*	Forward primer	GACCTGTACGCCAACACAGT	60 °C	129
	Reverse primer	CTCAGGAGGAGCAATGATCT		
*IL-1β*	Forward primer	TACGAATCTCCGACCACCA	60 °C	268
	Reverse primer	GGACCAGACATCACCAAGC		
*IL-6*	Forward primer	TACATCCTCGACGGCATCTC	60 °C	252
	Reverse primer	AGCTCTGGCTTGTTCCTCAC		
*IL-8*	Forward primer	GCTCTGTGTGAAGGTGCAGTTT	60 °C	144
	Reverse primer	TTCTGTGTTGGCGCAGTGT		
*IL-10*	Forward primer	CACCTTCCAGTGTCTC	60 °C	146
	Reverse primer	GGCTGGTTAGGAACTC		

*qRT-PCR* quantitative reverse-transcriptase PCR

### TUNEL and immunohistochemistry staining

We detected apoptosis cells by employing terminal deoxynucleotidyl transferase-mediated dUTP nick end-labeling (TUNEL) staining according to the manufacturer’s protocol (Vazyme). To detect the expression level of actived-caspase3 in kidney tissues and NRK-52E cells, we performed immunohistochemistry staining assay. After inactivating endogenous enzymes by 3% H_2_O_2_, the slices of kidney tissues and cells were incubated with actived-caspase3 antibody (1:50, Bioworld) overnight at 4 °C, then incubated with biotinylated sheep anti-rabbit IgG. The signal was developed by DAB staining and hematoxylin counterstaining.

### PDGF-DD enzyme-linked immunosorbent assay

PDGF-DD ELISA kit was purchased from Donglin Sci&Tech Development (China). PDGF-DD in the CdM was detected according to the operating instructions. The absorbance at 450 nm was measured and the corresponding concentration was calculated according to the standard curve.

### PDGF-DD siRNA transfection

PDGF-DD small interfering RNA (siRNA) and the matching scramble control siRNA (N.C) were purchased from GenePharma (Suzhou, China). We used Lipofectamine 2000 (Invitrogen) to transfect siRNA into hucMSCs according to the manufacturer’s instructions. The efficiency of PDGF-DD knockdown was evaluated through quantitative reverse-transcriptase PCR (qRT-PCR), western blotting, and enzyme-linked immunosorbent assay (ELISA). Experiments of PDGF-DD knockdown were divided in four groups: DMSO N.C (DMSO-hucMSCs treatment with N.C), Res N.C (Res-hucMSCs treatment with N.C), DMSO siRNA (DMSO-hucMSCs treatment with PDGF-DD siRNA), and Res siRNA (Res-hucMSCs treatment with PDGF-DD siRNA).

### Colony formation assay

hucMSCs (1 × 10^3^; with or without Res treatment) were seeded into six-well plates and incubated in 5% CO_2_ at 37 °C. The cells were replaced with fresh medium every 2 days. After incubation for 10 days, the cells were fixed with 4% paraformaldehyde for 30 min, then stained with 1% crystal violet for 10 min.

### Cell-counting assay

HUVEC cells were seeded at a density of 2 × 10^3^cells/well in 96-well plates for 24 h and replaced the medium with the CdM of hucMSCs (with or without Res treatment). Cells were counted at 24, 48, and 72 h, respectively, and each sample were repeated in triplicate. HUVEC cell proliferation could also be measured using a real-time cellular analysis system. HUVEC cells were seeded at a density of 2 × 10^3^ cells/well in a 16-well plate with electrodes, the CdM of DMSO-hucMSCs or Res-hucMSCs (100 μL) were added into the wells after cells culturing for 20 h, and continue to monitor for up to 65 h.

### Cell migration assay

HUVEC cells (5 × 10^4^) were suspended in serum-free high-glucose DMEM and seeded in the upper chamber, and the CdM of hucMSCs (with or without Res treatment) was placed in lower chamber. After incubation for 8 h, the cells that migrated through the membrane were fixed with 4% paraformaldehyde for 30 min, then stained with crystal violet for 10 min. The cells were observed by using a microscope (Nikon) and at least six fields of cells were assayed for each group.

### Immunofluorescence analysis

The previous steps were same as the immunohistochemistry staining assay. Primary antibody CD31 (1:50, Bioworld) were incubated overnight, followed by incubation with Cy3-labeled anti-rabbit IgG secondary antibody (1: 500, invitrogen) at 37 °C for 30 min. The nuclei were counterstained with Hoechst 33342 (1:200; Sigma-Aldrich).

### Statistical analysis

All data were shown as mean ± SD. Statistical analysis between groups was performed by GraphPad Prism 5.0 software (San Diego, USA). Statistical differences in multiple groups were determined by one-way analysis of variance followed by Tukey’s post tests. Statistical differences between two groups were determined by two-tailed paired Student’s *t*-test. The *P*-value < 0.05 was considered statistically significant.

## Results

### Res-hucMSCs exerted an improved repairing effect on kidney injury

**Fig. 1:**
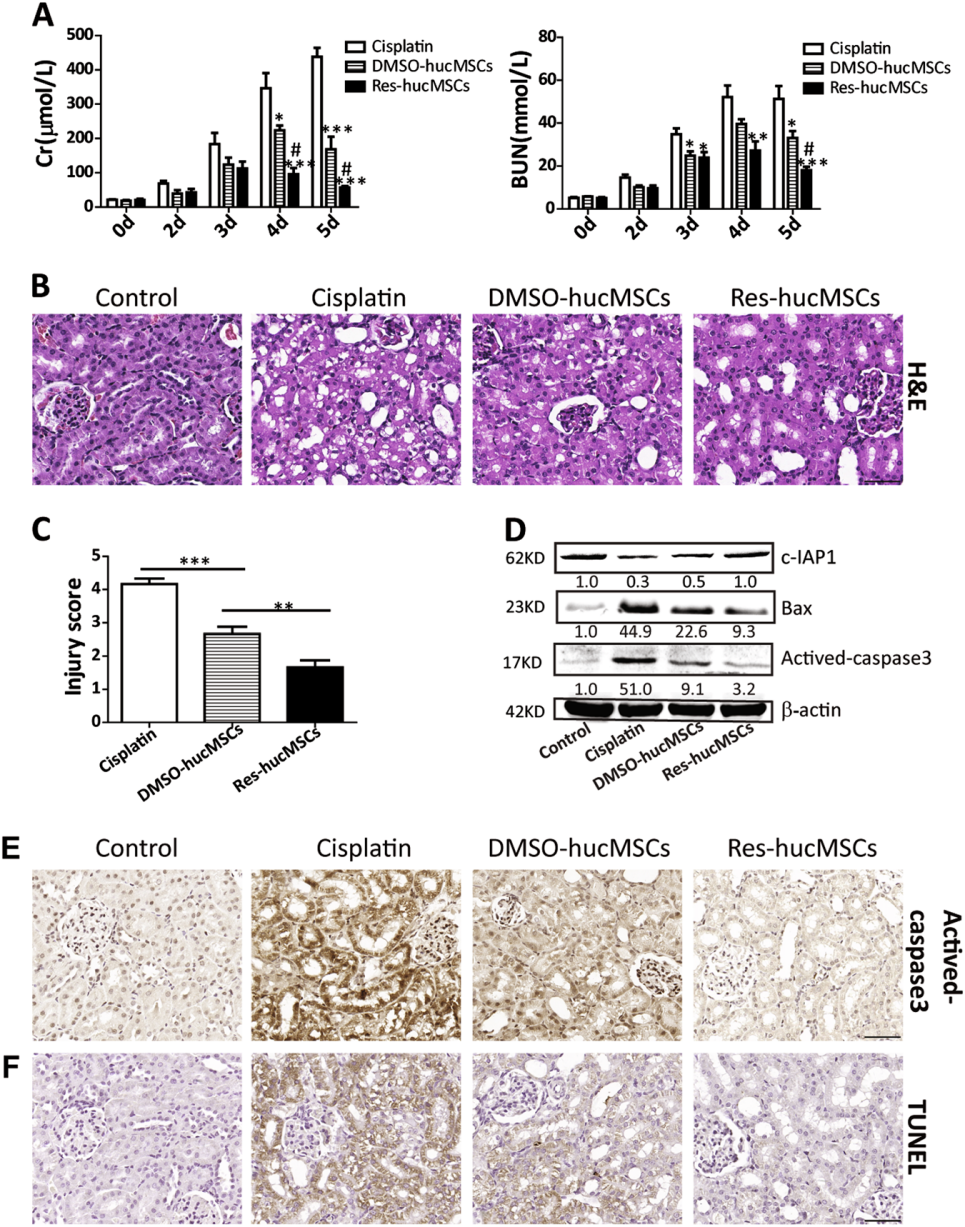
Res-hucMSCs exerted an improved repairing effect on kidney injury. **a** Serum Cr and BUN levels in cisplatin, DMSO-hucMSCs, and Res-hucMSCs groups (*P < 0.05, **P < 0.01, and ***P < 0.001 vs. cisplatin group, ^#^*P* < 0.05 vs. DMSO-hucMSCs group). **b** Representative micrographs of renal histology at Day 5 after cisplatin injection in the four groups (magnification × 200, Scale bars = 50 μm). **c** The degree of tubular damage was scored by choosing ten non-overlapping fields (magnification × 200) and calculating the percentage of tubules in the kidney cortex, which exhibited tubular cells necrosis and cast deposition as follows: 0, none; 1, ≤ 10%; 2, 10–25%; 3,.25–45%; 4, 45–75%; and 5, > 75%. The tubular injury score was calculated at Day 5 after cisplatin injection (**P < 0.01, ***P < 0.001). **d** Western blotting analysis of Bax, actived-caspase3, and c-IAP1 protein levels in the kidney tissues at Day 5 after cisplatin injection. Data are expressed as relative ratios of specific proteins to β-actin and shown as numbers under individual blots. **e** Immunohistochemical staining of actived-caspase3 in the cisplatin-induced injured kidney sections (magnification × 200, Scale bars = 50 μm). **f** Representative images of TUNEL staining in the kidney sections at Day 5 after cisplatin treatment (magnification × 200, Scale bars = 50 μm)

To define whether Res-husMSCs exert an improved repairing effect on kidney injury, we established cisplatin-induced AKI models and evaluated the repairing ability of Res-hucMSCs in AKI models. Results showed that serum Cr and BUN levels increased markedly at Day 3 and remained at a high level until Day 5 after cisplatin injection. Transplanting Res-hucMSCs significantly reduced serum Cr and BUN levels compared with DMSO-hucMSCs (Fig. 1a). Hematoxylin and eosin (H&E) staining of kidney tissues slices revealed that treating with DMSO-hucMSCs or Res-hucMSCs alleviated cisplatin-induced kidney injury as identified by fewer necrotic renal tubules and protein casts, and Res-hucMSCs was more effective than DMSO-hucMSCs in alleviating pathological injury (Fig. 1b). In addition, histological injure score was obviously reduced in the DMSO-hucMSCs or the Res-hucMSCs group compared with the cisplatin group, and the injury score was lowest in the Res-hucMSCs group (Fig. 1c). Western blotting detected the expression of apoptosis-associated proteins. Results showed that transplanting DMSO-hucMSCs or Res-hucMSCs decreased the expression of Bax and actived-caspase3, increased the expression of c-IAP1 and the anti-apoptosis effect of Res-hucMSCs was more obvious than did DMSO-hucMSCs (Fig. 1d). Immunohistochemical staining further confirmed the expression of actived-caspase3, and the average percent of actived-caspase3-positive cells in control, cisplatin, DMSO-hucMSCs, and Res-hucMSCs groups was 32.6%, 94%, 83.8%, and 21.2% (Fig. 1e), respectively. Furthermore, TUNEL assay indicated that a reduced number of apoptotic cells in both DMSO-hucMSCs and Res-hucMSCs groups, and the Res-hucMSCs group had fewer apoptotic cells than did DMSO-hucMSCs group. The average percent of TUNEL-positive cells in control, cisplatin, DMSO-hucMSCs, and Res-hucMSCs groups was 1%, 76.6%, 20%, and 2.5% (Fig. 1f), respectively. Altogether, Res-hucMSCs showed a more efficient repair ability than did hucMSCs in cisplatin-induced kidney injury.

### Effect of Res-hucMSCs on NRK-52E cells apoptosis

**Fig. 2:**
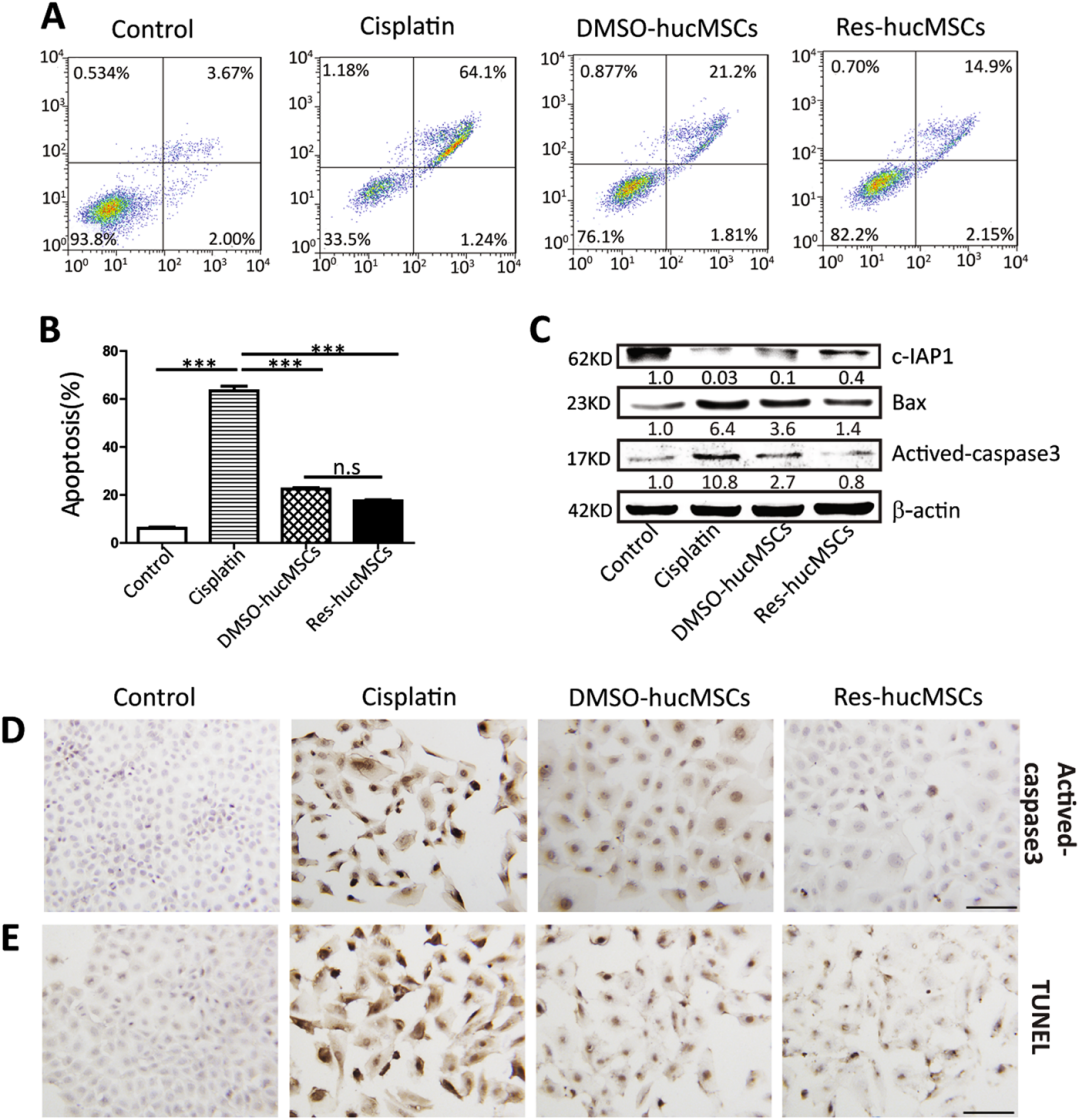
Effect of Res-hucMSCs on NRK-52E cells apoptosis. **a** Flow cytometry detected apoptotic NRK-52E cells. **b** The statistical figure of apoptotic NRK-52E cells (***P < 0.001). **c** Western blotting quantification of Bax, actived-caspase3, and c-IAP1 expression in NRK-52E cells. **d** Immunohistochemical staining of actived-caspase3 in cisplatin-induced injured NRK-52E cells with or without DMSO-hucMSCs and Res-hucMSCs treatment (magnification × 200, Scale bars = 50 μm). **e** Representative images of TUNEL staining in cisplatin-induced injured NRK-52E cells with or without DMSO-hucMSCs and Res-hucMSCs treatment (magnification × 200, Scale bars = 50 μm)

To confirm the effect of Res-hucMSCs in vitro, we used 7.5 μmol/L of cisplatin-treated NRK-52E cells. After treating with cisplatin for 12 h, there was a significantly increased number of apoptotic NRK-52E cells. By contrast, treatment with DMSO-hucMSCs or Res-hucMSCs could effectively reverse cisplatin-induced NRK-52E cells apoptosis and the number of apoptotic NRK-52E cells was least in the Res-hucMSCs group (Fig. 2a). The statistical figure was shown in Fig. 2b. The expression of apoptosis-associated proteins was tested by western blotting. Results showed that the expression of Bax and actived-caspase3 significantly increased, whereas the expression of c-IAP1 significantly decreased after cisplatin treatment. Nevertheless, changes in the expression of apoptosis-associated proteins could be rescued in DMSO-hucMSCs or Res-hucMSCs group. In the Res-hucMSCs group, the expression levels of Bax and actived-caspase3 were lowest and the expression level of c-IAP1 was highest (Fig. 2c). Immunohistochemical staining further confirmed the expression of actived-caspase3 and the result was consistent with that by western blotting. The average percent of actived-caspase3-positive cells in control, cisplatin, DMSO-hucMSCs, and Res-hucMSCs groups was 3.8%, 86.7%, 62.2%, and 14.3% (Fig. 2d), respectively. Furthermore, TUNEL assay showed that the number of TUNEL-positive cells obviously increased after cisplatin treatment, whereas the number of TUNEL-positive cells decreased in both DMSO-hucMSCs and Res-hucMSCs groups. Res-hucMSCs group had fewer TUNEL-positive cells than did DMSO-hucMSCs group. The average percent of TUNEL-positive cells in control, cisplatin, DMSO-hucMSCs, and Res-hucMSCs groups was 9.2%, 93.2%, 77%, and 40.9% (Fig. 2e), respectively. In summary, Res-hucMSCs could significantly inhibit cisplatin-induced NRK-52E cells apoptosis compared with hucMSCs.

### Res regulated hucMSCs proliferation and apoptosis via promoting PDGF-DD autocrine in hucMSCs

**Fig. 3:**
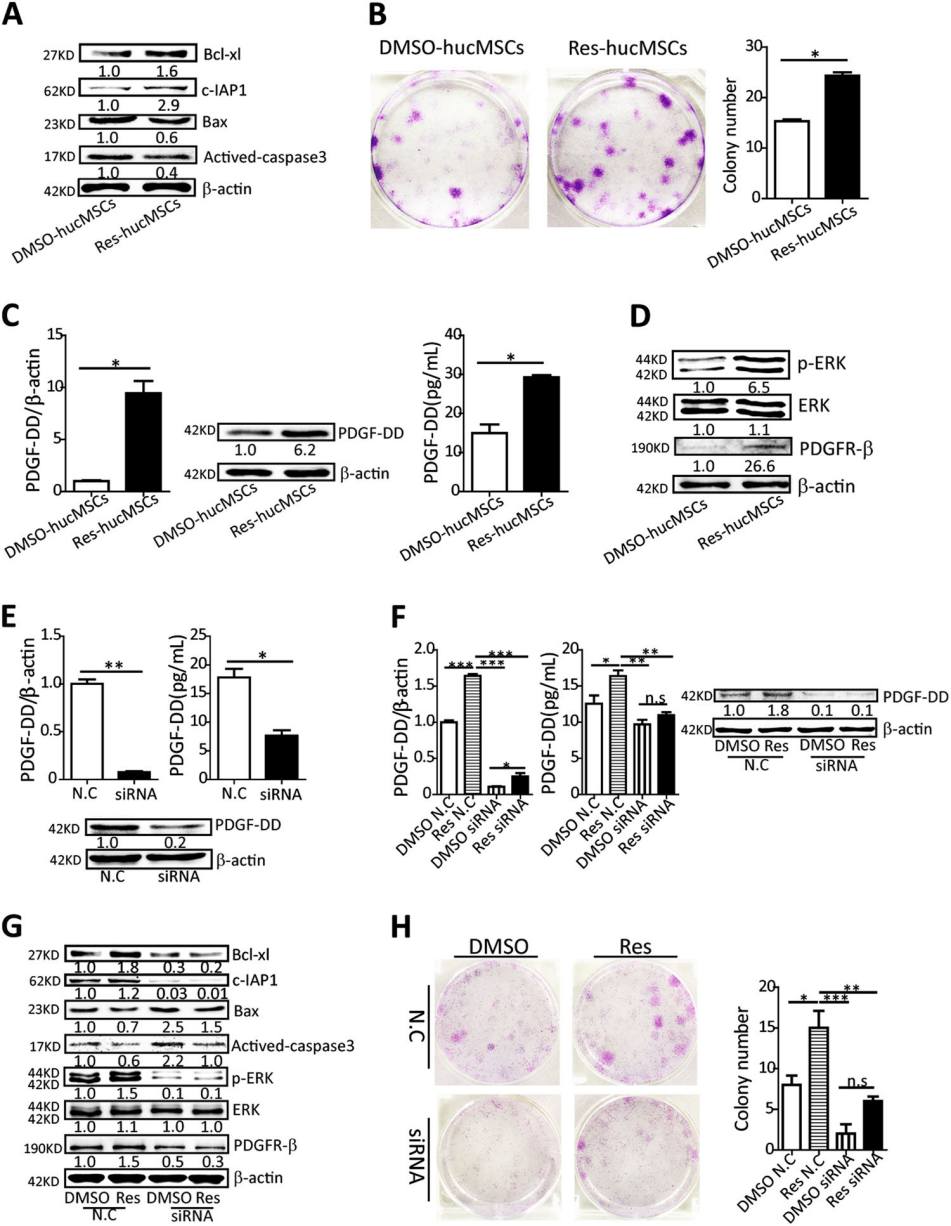
Resveratrol regulated hucMSCs proliferation and apoptosis via promoting PDGF-DD autocrine in hucMSCs. **a** Expression of Bax, actived-caspase3, c-IAP1, and Bcl-xl in DMSO-hucMSCs or Res-hucMSCs were measured by western blotting. **b** Colony-forming assay for hucMSCs with or without resveratrol treatment (*P < 0.05). **c** The PDGF-DD level in hucMSCs with or without resveratrol treatment were measured by qRT-PCR, western blotting, and ELISA assay (*P < 0.05). **d** Expression of PDGFR-β, p-ERK, and ERK in DMSO-hucMSCs or Res-hucMSCs were detected by western blotting. **e** Verified the efficiency of PDGF-DD knockdown by using qRT-PCR, western blotting, and ELISA assay (*P < 0.05 and **P < 0.01). **f** Resveratrol treatment did not rescue the decrease of PDGF-DD levels induced by the PDGF-DD siRNA (*P < 0.05, **P < 0.01, ***P < 0.001). **g** Western blotting for the expression of Bax, actived-caspase3, c-IAP1, Bcl-xl, PDGFR-β, p-ERK, and ERK in DMSO-hucMSCs and Res-hucMSCs with or without PDGF-DD siRNA treatment. **h** Colony-forming assay for DMSO-hucMSCs and Res-hucMSCs with or without PDGF-DD siRNA treatment (*P < 0.05, **P < 0.01 and ***P < 0.001)

A hostile microenvironment of the injured tissues including oxidative stress, inflammatory response, and development of pro-apoptotic factors induces implanted hucMSCs apoptosis, which blur the efficacy of hucMSCs-based therapy^19,20^. Therefore, we tested the protective effect of Res on hucMSCs. Results showed that Res decreased actived-caspase3 and Bax protein levels, increased c-IAP1 and Bcl-xl protein levels in hucMSCs, and enhanced the colony-forming ability of hucMSCs (Fig. 3a, b). qRT-PCR assay showed that Res had no effect on the expression of inflammatory factors in hucMSCs (Figure S1). Next, we investigated the underlying mechanisms about the protective effect of Res on hucMSCs from the perspective of stemness or paracrine of hucMSCs. Results showed that Res had no effect on the expression of stemness transcription factors and majority of cytokines in hucMSCs (Figure S2), whereas it increased the expression and secretion of PDGF-DD in hucMSCs (Fig. 3c). Meanwhile, the PDGF receptor-β and PDGF-DD/PDGFR downstream p-ERK levels were higher in Res-hucMSCs than in DMSO-hucMSCs (Fig. 3d). To confirm the role of PDGF-DD in the effect of Res on hucMSCs, we used the PDGF-DD siRNA to knock down the PDGF-DD expression in hucMSCs and verified the efficiency by using both mRNA and protein levels (Fig. 3e). Res did not rescue the decrease of PDGF-DD levels induced by the PDGF-DD siRNA (Fig. 3f). PDGF-DD knockdown decreased the expression of PDGFR-β in Res-hucMSCs, correlating with inhibiting ERK signal pathway, which increased the level of Bax and actived-caspase3, decreased the level of c-IAP1 and Bcl-xl in Res-hucMSCs, and weakened the colony formation ability of Res-hucMSCs (Fig. 3g, h). In addition, nude mice injected subcutaneously with Res-hucMSCs for 4 weeks did not form tumors (Figure S3), which ensured the safety of Res pre-treatment. Collectively, Res regulated hucMSCs proliferation and apoptosis via promoting PDGF-DD autocrine in hucMSCs.

### Res-hucMSCs exerted an improved renal protective effect via paracrine PDGF-DD

**Fig. 4:**
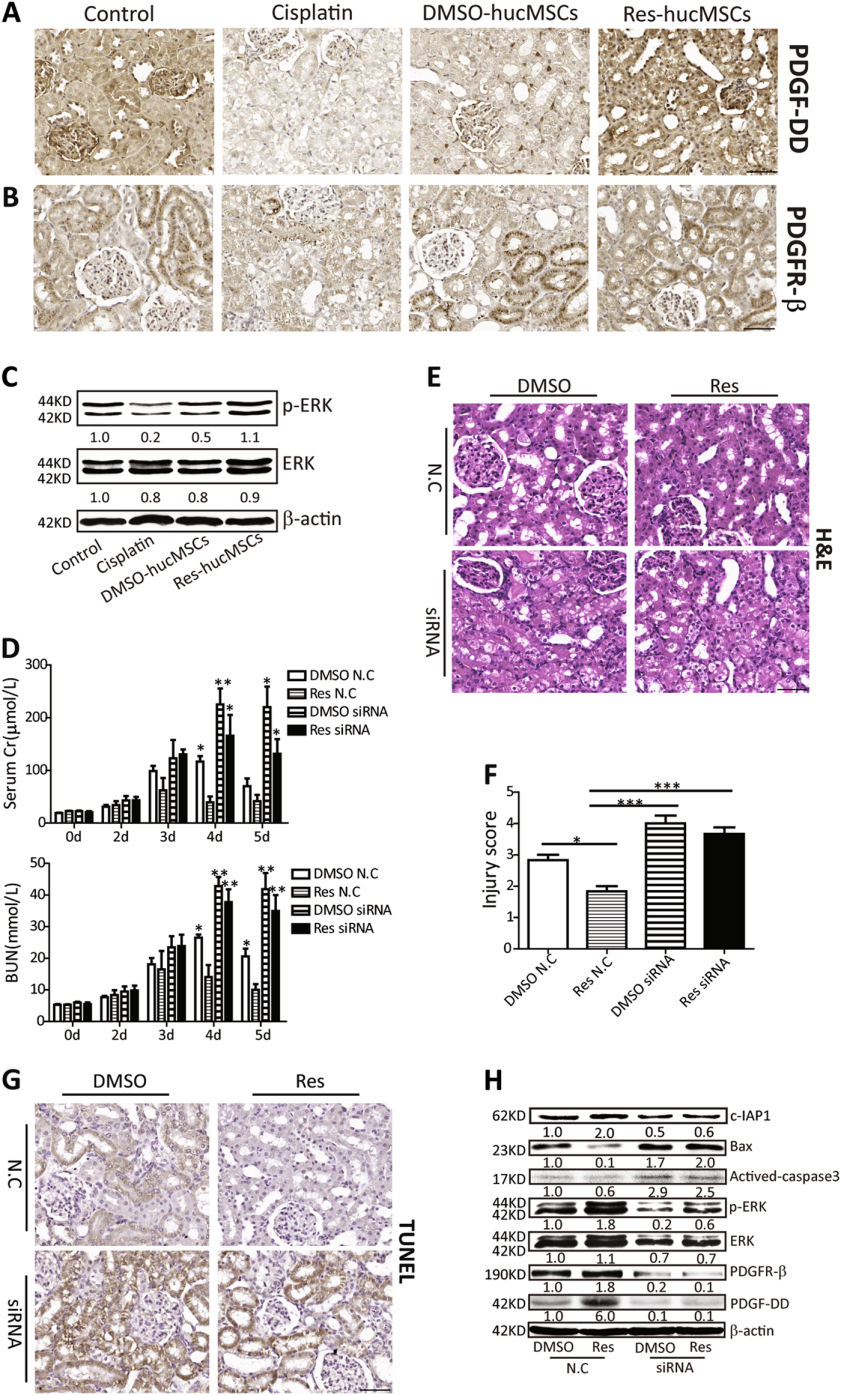
Res-hucMSCs exerted an improved renal protective effect via paracrine PDGF-DD. **a** Cisplatin-induced kidney injury models were treated with PBS, DMSO-hucMSCs, and Res-hucMSCs. The expression of PDGF-DD were measured by immunohistochemical staining (magnification × 200, Scale bars = 50 μm). **b** Immunohistochemical staining of PDGFR-β in kidney tissues at Day 5 after ciaplatin injection (magnification × 200, Scale bars = 50 μm). **c** Western blotting quantification of p-ERK and ERK expression in control, cisplatin, DMSO-hucMSCs, and Res-hucMSCs groups. **d** After cisplatin-induced injured kidney was treated with Res-hucMSCs with or without PDGF-DD siRNA treatment, serum Cr and BUN levels were measured (*P < 0.05 and **P < 0.01 vs. Res N.C group). **e** The cisplatin-induced kidney injury models were treated with Res-hucMSCs with or without PDGF-DD siRNA treatment for 4 days, the kidney tissues were subjected to H&E staining (magnification × 200, Scale bars = 50 μm). **f** Tubular injury score was calculated after different treatment (*P < 0.05 and ***P < 0.001). **g** The number of apoptotic cells in kidney tissues were determined using TUNEL staining (magnification × 200, Scale bars = 50 μm). **h** Western blotting for the expression of Bax, actived-caspase3, c-IAP1, PDGF-DD, PDGFR-β, p-ERK, and ERK in injured kidney tissues after different treatment

As shown in Figure S4, the ability of Res-hucMSCs homing to kidney tissues was significantly superior to that of DMSO-hucMSCs. Moreover, the number of Res-hucMSCs homing in kidney tissues was still a lot at 96 h, whereas DMSO-hucMSCs homing in kidney tissues was almost absent at 96 h. These results showed that Res pre-treatment promoted the engraftment of infused hucMSCs in kidney tissues and protected infused hucMSCs against a hostile microenvironment. The protective effect of Res on hucMSCs increased the survival rate of hucMSCs and may encourage hucMSCs to secrete more PDGF-DD in vivo. Thus, we detected the level of PDGF-DD in kidney tissues after different treatment. Results showed that when compared the DMSO-hucMSCs or Res-hucMSCs group with the cisplatin group, the PDGF-DD level obviously increased and the PDGF-DD level was highest in the Res-hucMSCs group. The average percent of PDGF-DD-positive cells in control, cisplatin, DMSO-hucMSCs, and Res-hucMSCs groups was 53.3%, 9.5%, 20.3%, and 85% (Fig. 4a), respectively. The level of PDGFR-β in the Res-hucMSCs group was higher than in the DMSO-hucMSCs group. The average percent of PDGFR-β-positive cells in control, cisplatin, DMSO-hucMSCs, and Res-hucMSCs groups was 55.4%, 32%, 53%, and 76.3% (Fig. 4b), respectively. Furthermore, Res-hucMSCs more effectively activated the ERK pathway in renal tubular cells than did DMSO-hucMSCs (Fig. 4c). To assess the role of PDGF-DD in the renal protection of Res-hucMSCs, we injected Res-hucMSCs with N.C or PDGF-DD siRNA into cisplatin-induced AKI rat models. As expected, Res-hucMSCs with N.C exhibited a decent recovery, whereas Res-hucMSCs with PDGF-DD siRNA remained incompletely repaired. Compared with the Res N.C group, we observed a significant rise in serum Cr and BUN levels in the Res siRNA group (Fig. 4d). H&E staining of kidney tissue slices indicated that the Res N.C group had fewer necrotic renal tubules and protein casts than did the Res siRNA group (Fig. 4e). Moreover, histological injure score was markedly higher in the Res siRNA group than in the Res N.C group (Fig. 4f). In addition, the Res N.C group had fewer apoptotic cells than did the Res siRNA group. The average percent of TUNEL-positive cells in DMSO N.C, Res N.C, DMSO siRNA, and Res siRNA groups was 15%, 3%, 71.4%, and 51% (Fig. 4g), respectively. Moreover, compared with the Res N.C group, the Bax and actived-caspase3 level significantly increased, whereas the c-IAP1 level significantly decreased in Res siRNA group. Western blotting further indicated a significant decrease of the PDGF-DD and PDGFR-β levels in the Res siRNA group, which inhibited the ERK signal pathway in renal tubular cells (Fig. 4h). In brief, Res-hucMSCs exerted a more effective renal protective effect than hucMSCs via promoting PDGF-DD paracrine.

### Res-hucMSCs promoted angiogenesis via paracrine PDGF-DD

**Fig. 5:**
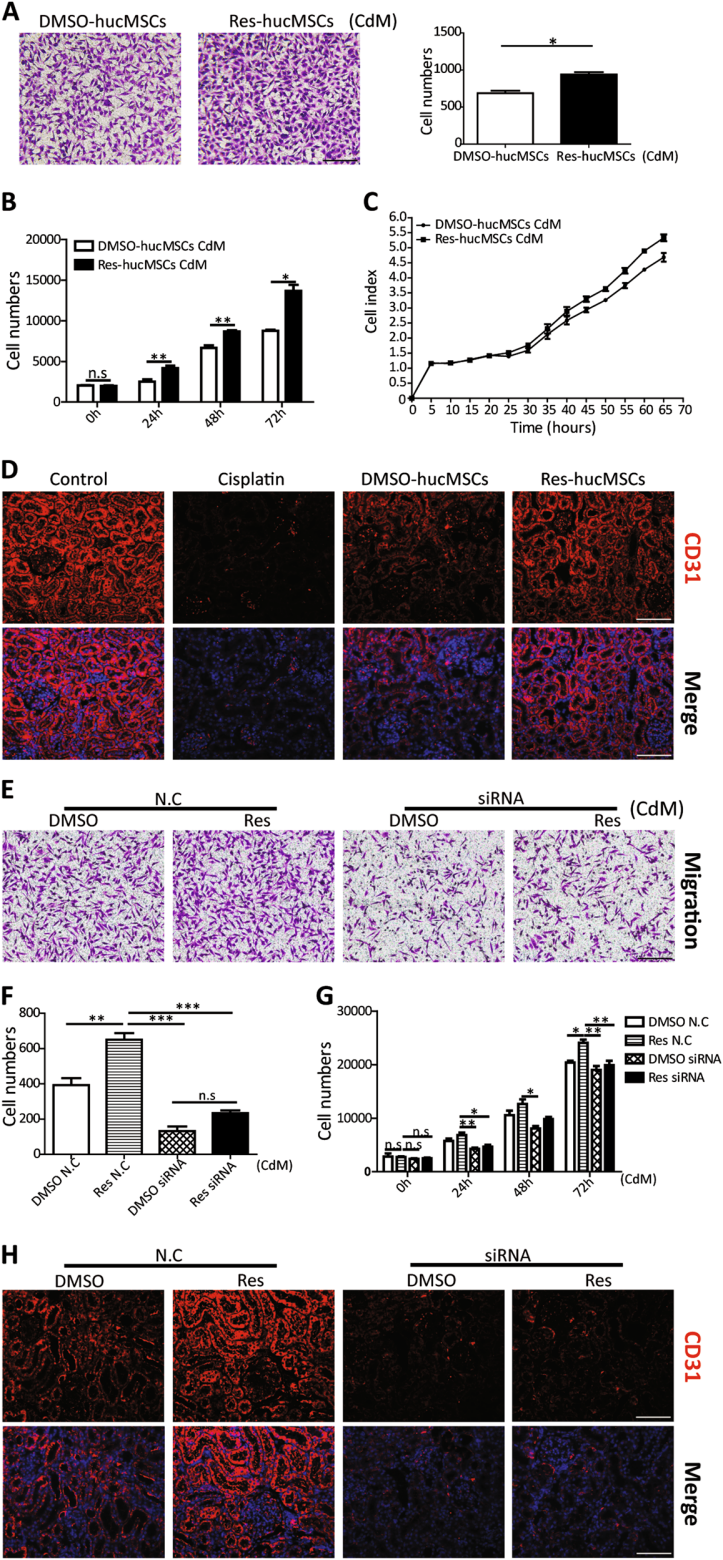
Res-hucMSCs promoted angiogenesis via paracrine PDGF-DD. **a** The CdM of Res-hucMSCs promoted migration of HUVEC cells compared with the CdM of DMSO-hucMSCs (*P < 0.05). **b** Cell-counting assay for the proliferation of HUVEC cells (*P < 0.05 and **P < 0.01). **c** HUVEC cells proliferation was measured using a real-time cellular analysis (RTCA) system. **d** Representative immunofluorescence images of CD31 expression in injured kidney tissues after different treatment (magnification × 200, Scale bars = 50 μm). **e** The migration ability of HUVEC cells treated with the CdM of Res-hucMSCs with or without PDGF-DD siRNA treatment. **f** The statistical figure of migratory HUVEC cells (**P < 0.01 and ***P < 0.001). **g** Cell-counting assay for the proliferation of HUVEC cells treated with the CdM of Res-hucMSCs with or without PDGF-DD siRNA treatment (*P < 0.05 and **P < 0.01). **h** The cisplatin-induced kidney injury models were treated with Res-hucMSCs with or without PDGF-DD siRNA treatment for 4 days. The expression of CD31 in the injured kidney area was measured using immunofluorescence staining (magnification × 200, Scale bars = 50 μm)

Besides regulating cell progression, PDGF-DD has an important role in angiogenesis^21^. Angiogenesis is a crucial mechanism in injured tissues repair^22,23^. Therefore, we came up with a hypothesis: angiogenesis may participate in the renal protective effects of Res-hucMSCs. Collect the CdM of hucMSCs with or without Res treatment and observe their effects on HUVEC cells functions. The CdM of Res-hucMSCs enhanced the HUVEC cells migration and proliferation ability compared with the CdM of DMSO-hucMSCs (Fig. 5a–c). Meanwhile, immunofluorescence staining assay showed that the expression of CD31, a marker of vascular endothelial cell highly increased in the Res-hucMSCs group compared with that in the DMSO-hucMSCs group in vivo (Fig. 5d). To verify whether PDGF-DD was involved in angiogenesis, we collected the CdM of Res-hucMSCs with or without PDGF-DD siRNA treatment and evaluated the effect of those CdM on HUVEC functions. Not unexpectedly, the CdM of Res-hucMSCs with PDGF-DD siRNA inhibited the migration and proliferation of HUVEC cells compared with the CdM of Res-hucMSCs with N.C (Fig. 5e–g). Furthermore, the results of immunofluorescence staining showed that PDGF-DD knockdown reduced the expression of CD31 in vivo (Fig. 5h). The above results displayed that angiogenesis induced by PDGF-DD was involved in renal protective effects of Res-hucMSCs.

## Discussion

MSCs are a promising therapeutic tool in regenerative medicine due to their self-renewal and multi-directional differentiation potency. A large number of studies showed that MSCs could repair cisplatin-induced AKI^24^, ischemia/reperfusion-induced acute renal failure^25^, and unilateral ureteral obstruction-induced renal fibrosis^26^. Nevertheless, the vast majority of transplanted MSCs do not survive for a long time and only a very small number of survived MSCs home to damaged kidney in vivo. The limited survival and engraftment of transplanted MSCs blur the effectiveness of MSCs-based therapy^27^. Therefore, to maximize the clinical utility of MSCs, strategies must be employed to improve their therapy efficacy.

One of the common strategies that improve the therapeutic effects of stem cell transplantation is pre-treatment stem cells with small molecules drugs. Compared with genetic manipulation, small-molecule drugs have a prominent advantage: their effects can be fine-tuned by altering their working concentration, duration time, and compositions^28^. Melatonin efficiently prevented the stemness loss of bone marrow mesenchymal stem cells (BMMSCs) and improved their therapeutic efficacy in bone repair and immunological colitis^29^. Osthole^30^ enhanced the osteogenic ability of periodontal ligament stem cells derived from periodontitis tissues, which could be a potential strategy to treat periodontitis. These studies indicate that small-molecule drugs are potential candidates for stem cell manipulation in regenerative medicine.

Res, a natural small-molecule compound, is a phytophenol with multifarious properties and possesses therapeutic activities in various diseases^31–33^. Res generally acknowledged as anti-aging or stem cell-protection substances, which promoted MSCs proliferation, maintained differentiation potential of MSCs, and delayed MSCs senescence^34,35^. Co-administration of Res and MSCs could augment therapeutic efficiency of MSCs in autoimmune encephalomyelitis^36^ and cardiomyopathy^37^, suggesting improving effects of Res infusion on MSCs-based therapy. However, the role of Res pre-treatment in MSCs-based therapy has not been characterized. In this study, we found that Res-hucMSCs improved renal function compared with DMSO-hucMSCs, as demonstrated by the decrease of serum Cr and BUN levels, as well as the decrease of histological injury score and apoptotic kidney tubular cells. HucMSCs primed with Res exhibited more effective repair effects than untreated hucMSCs in cisplatin-induced AKI models. Res pre-treatment is safe, efficient and low cost, which is expected to emerge as a promising strategy to improve MSCs-based therapy.

Protection of the implanted cells from the adverse environment of the injured tissues is of utmost importance for a successful cell therapy. Administration of Res together with MSCs positively contributes to the number of living MSCs in injured liver^38^. Here we confirmed that Res enhanced the proliferation and anti-apoptosis ability of hucMSCs via promoting PDGF-DD autocrine in hucMSCs, which protected hucMSCs against a hostile microenvironment to survive for a long time. PDGF-DD, a recently discoverable member of the PDGF family, binds to PDGF receptor-β with high affinity. PDGF-DD binds and activates PDGFR-β resulting in downstream phosphorylation of ERK^39^. The ERK signal pathway is involved in regulating proliferation and apoptosis process of cells. Our previous study indicated that exosomes derived from hucMSCs protected against cisplatin-induced renal tubular cells apoptoiss by activating ERK signal pathway^18^. In this study, Res-hucMSCs secreted more PDGF-DD into renal tubular cells, which activated ERK pathway and inhibited renal tubular cells apoptosis, eventually exerted a more effective repair ability than did hucMSCs in AKI.

Increasing evidences support the idea that MSCs exert a therapeutic effect via promoting angiogenesis^40–42^. PDGF-DD, an important angiogenic factor, can promote angiogenesis^43^. Angiogenesis involves a series of coordinated events, including degradation of the extracellular matrix around the vessel, proliferation, and migration of vascular endothelial cells and mural cells to assemble the new vessel, lumen formation, and pericytes and smooth muscle cells to construct the mural cell layer of the vessel wall^44^. Here, although it had no significant effect on tube-like structure formation of HUVEC cells, the CdM of Res-hucMSCs effectively stimulated proliferation and migration of HUVEC cells. Proliferation and migration of endothelial cells are an important link for angiogenesis. Furthermore, CD31 was in a high-level expression in the Res-hucMSCs group compared with that in the DMSO-hucMSCs group in vivo. In contrast, angiogenesis induced by Res-hucMSCs were abolished when Res-hucMSCs were treated with the PDGF-DD siRNA. All of these findings suggested that angiogenesis induced by PDGF-DD was involved in the renal protective effect of Res-hucMSCs.

We can roughly conclude from results of the present study that Res-hucMSCs protected against kidney injury by secreting PDGF-DD to activate ERK pathway in renal tubular cells and promote angiogenesis in endothelial cells. The mechanism of how Res increase PDGF-DD secretion in hucMSCs, however, is not clear. Nuclear factor E2-related factor 2 (Nrf2) is a master transcriptional regulator of cellular defenses against oxidative stress. Res significantly increased the Nrf2 expression in hucMSCs (Figure S5), which suggest that Res-hucMSCs is more resistant to cisplatin-induced oxidative stress microenvironment than hucMSCs. Furthermore, Malhotra et al.^45^ found that PDGF-C was a direct transcriptional target of Nrf2. In addition, BMMSCs stimulated by pro-inflammatory cytokines increased the expression of PDGF via the Nrf2-HIF-1α pathway and promoted prostate cancer growth^46^. Based on Figure S5, we suggest that Res promote the PDGF-DD expression of hucMSCs possibly through regulating Nrf2. On the other hand, Res is a known activator of sirtuin1 (SIRT1). Our study also confirmed that the SIRT1 level in hucMSCs obviously increased after Res treatment (Figure S5). Although the studies of SIRT1 regulating PDGF have not been reported yet, the studies of SIRT1 regulating other growth factors such as vascular endothelial growth factor and insulin-like growth factor have been endless^47–49^. Hence, SIRT1 may be involved in Res enhancing hucMSCs PDGF-DD secretion. Exosomes derived from hucMSCs act as transporters in cell–cell communication to deliver bioactive molecules from original cells to the recipient cells. PDGF-DD secreted by hucMSCs or Res-hucMSCs was transported through exosomes, which is unclear. All these assumptions need to be confirmed by further studies.

## Conclusion

Our results have clearly demonstrated that Res-modified hucMSCs secrete PDGF-DD to activate ERK pathway in renal tubular cells and promote angiogenesis in endothelial cells, which eventually have a higher efficiency than hucMSCs in the repair of cisplatin-induced kidney injury (Pattern diagram). This study provides a new therapeutic strategy to improve kidney function for patients with AKI.

## Electronic supplementary material

Figure S1

Figure S2

Figure S3

Figure S4

Figure S5

Supplementary figure legends
