# Supplementary figures and images for: Resveratrol improves human umbilical cord-derived mesenchymal stem cells repair for cisplatin-induced acute kidney injury
Source: Cell Death Dis. 2018 Sep 20;9(10):965. doi: 10.1038/s41419-018-0959-1 (PMC6148224; doi:10.1038/s41419-018-0959-1)

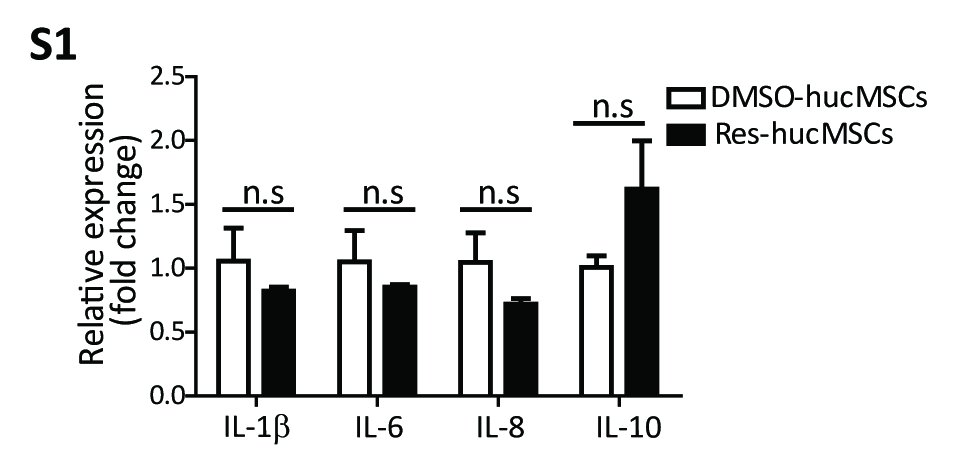

Supplement: Supplementary file 1 — Figure S1 [file 41419_2018_959_MOESM1_ESM.tif]

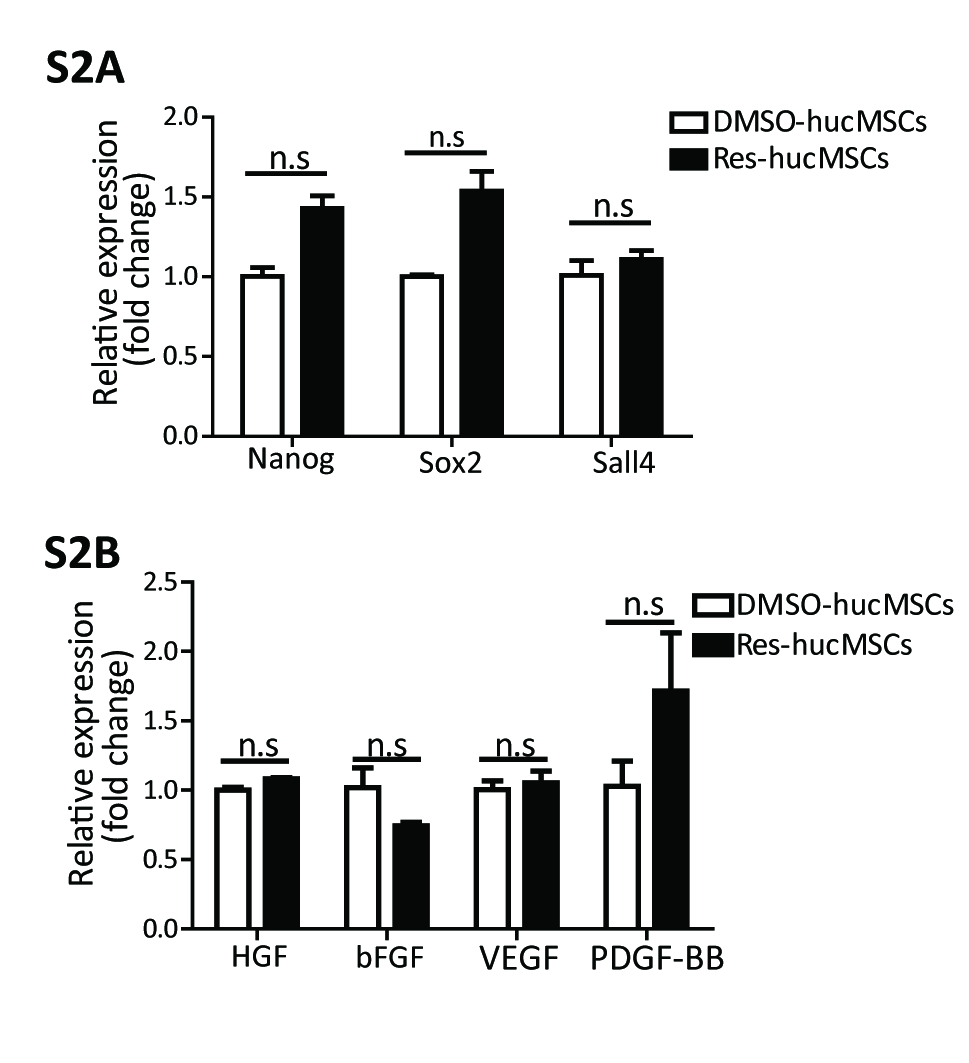

Supplement: Supplementary file 2 — Figure S2 [file 41419_2018_959_MOESM2_ESM.tif]

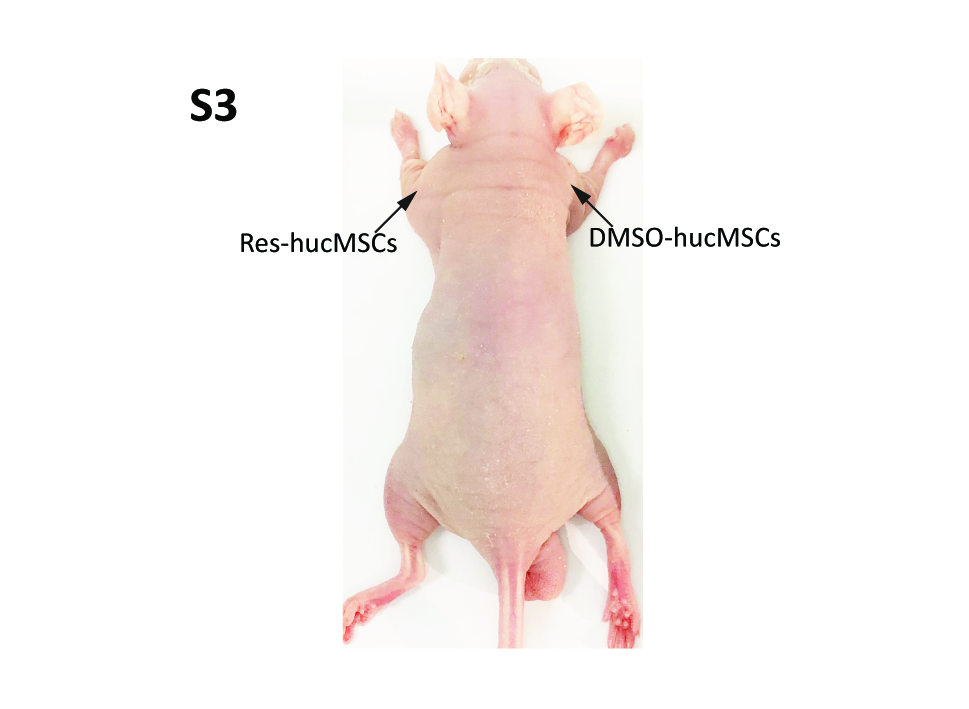

Supplement: Supplementary file 3 — Figure S3 [file 41419_2018_959_MOESM3_ESM.tif]

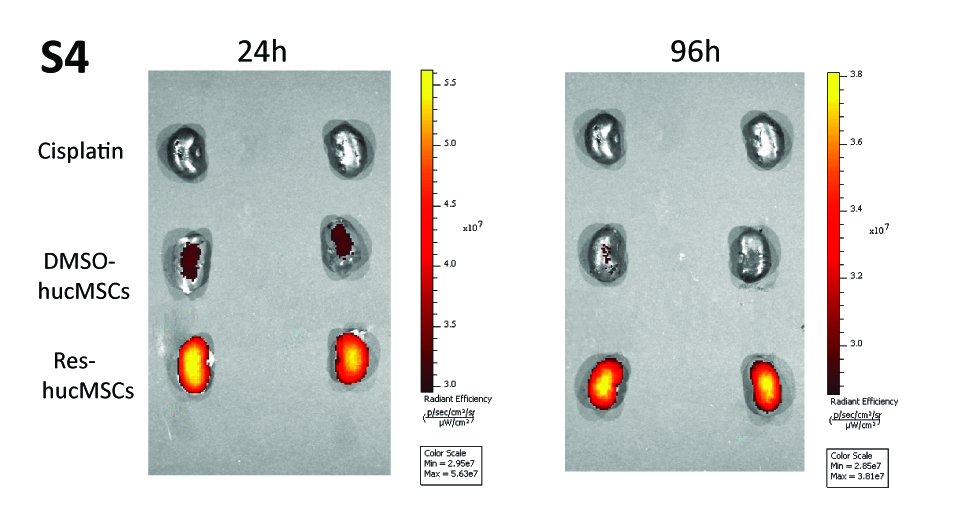

Supplement: Supplementary file 4 — Figure S4 [file 41419_2018_959_MOESM4_ESM.tif]

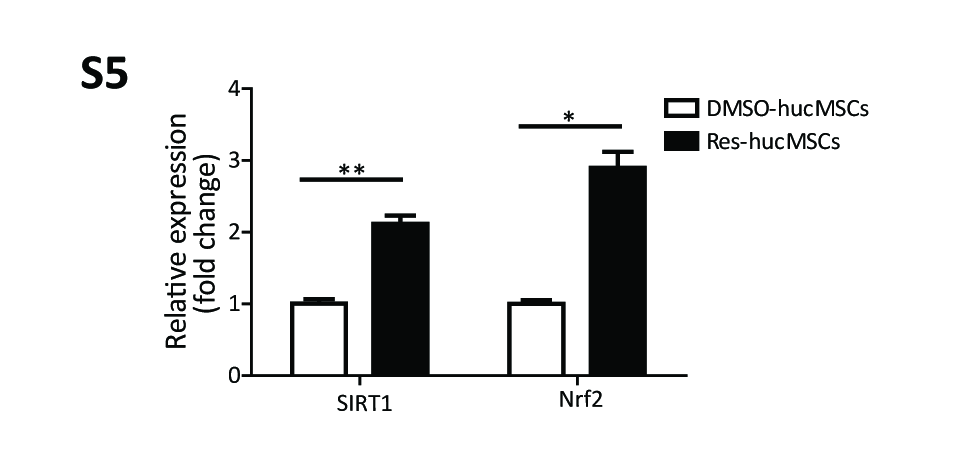

Supplement: Supplementary file 5 — Figure S5 [file 41419_2018_959_MOESM5_ESM.tif]
