# Supplementary material for: Resveratrol improves human umbilical cord-derived mesenchymal stem cells repair for cisplatin-induced acute kidney injury
Source: Cell Death Dis. 2018 Sep 20;9(10):965. doi: 10.1038/s41419-018-0959-1 (PMC6148224; doi:10.1038/s41419-018-0959-1)
Supplement: Supplementary file 6 — Supplementary figure legends [file 41419_2018_959_MOESM6_ESM.doc]

**Supplementary figure legends**

**Figure S1 Effect of resveratrol on inflammatory factors in hucMSCs.** QRT-PCR analysis of inflammatory factors levels in hucMSCs with or without resveratrol treatment.

**Figure S2 Effect of resveratrol on hucMSCs stemness and paracrine effect.** (A) QRT-PCR for stemness transcription factors such as Nanog, Sox2 and Sall4 in hucMSCs with or without resveratrol treatment. (B) QRT-PCR for cytokines which are closely related to the treatment of kidney injury in hucMSCs with or without resveratrol treatment.

**Figure S3 Safety evaluation of resveratrol pre-treatment.** 4 weeks after subcutaneous injection of Res-hucMSCs into nude mice, the oncogenic activity was evaluated.

**Figure S4 The engraftment of infused MSCs in renal tissues with and without resveratrol pre-treatment.** In-Vivo Imaging System was used to observe the engraftment of infused DMSO-hucMSCs or Res-hucMSCs which were labeled with DiR in renal tissues.

**Figure S5 Effect of resveratrol on SIRT1 and Nrf2 expression in hucMSCs.** QRT-PCR detected the SIRT1 and Nrf2 levels in hucMSCs with or without resveratrol treatment (**P*<0.05 and ***P*<0.01).
